# Supplementary figures and images for: Diagnostic accuracy of adenosine deaminase for pleural tuberculosis in a low prevalence setting: A machine learning approach within a 7-year prospective multi-center study
Source: PLoS One. 2021 Nov 4;16(11):e0259203. doi: 10.1371/journal.pone.0259203 (PMC8568264; doi:10.1371/journal.pone.0259203)

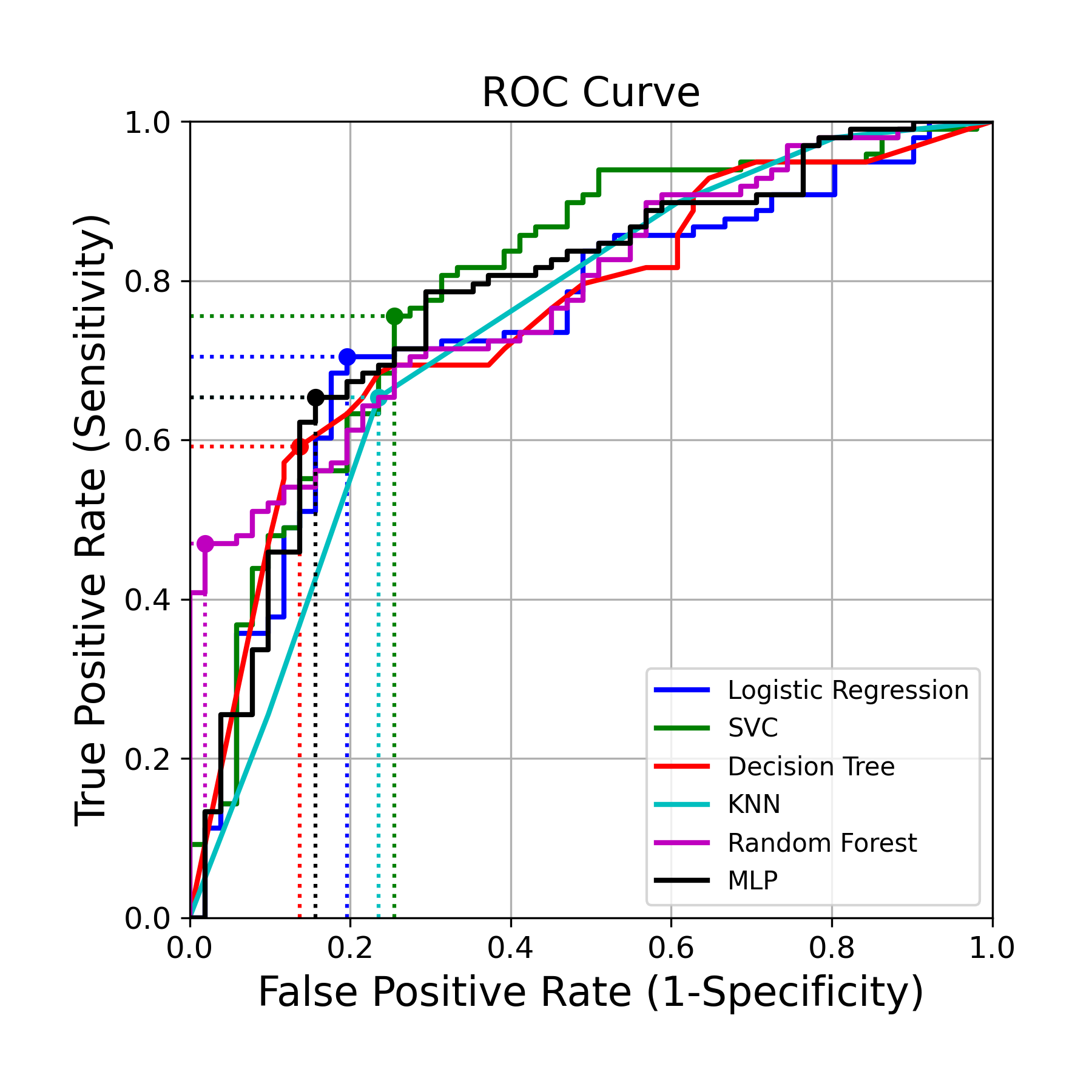

Supplement: S1 Fig — All training samples have been used except the ones identified as Tuberculous. The dots correspond to the points that maximize the Youden index. (TIF) [file pone.0259203.s001.tif]

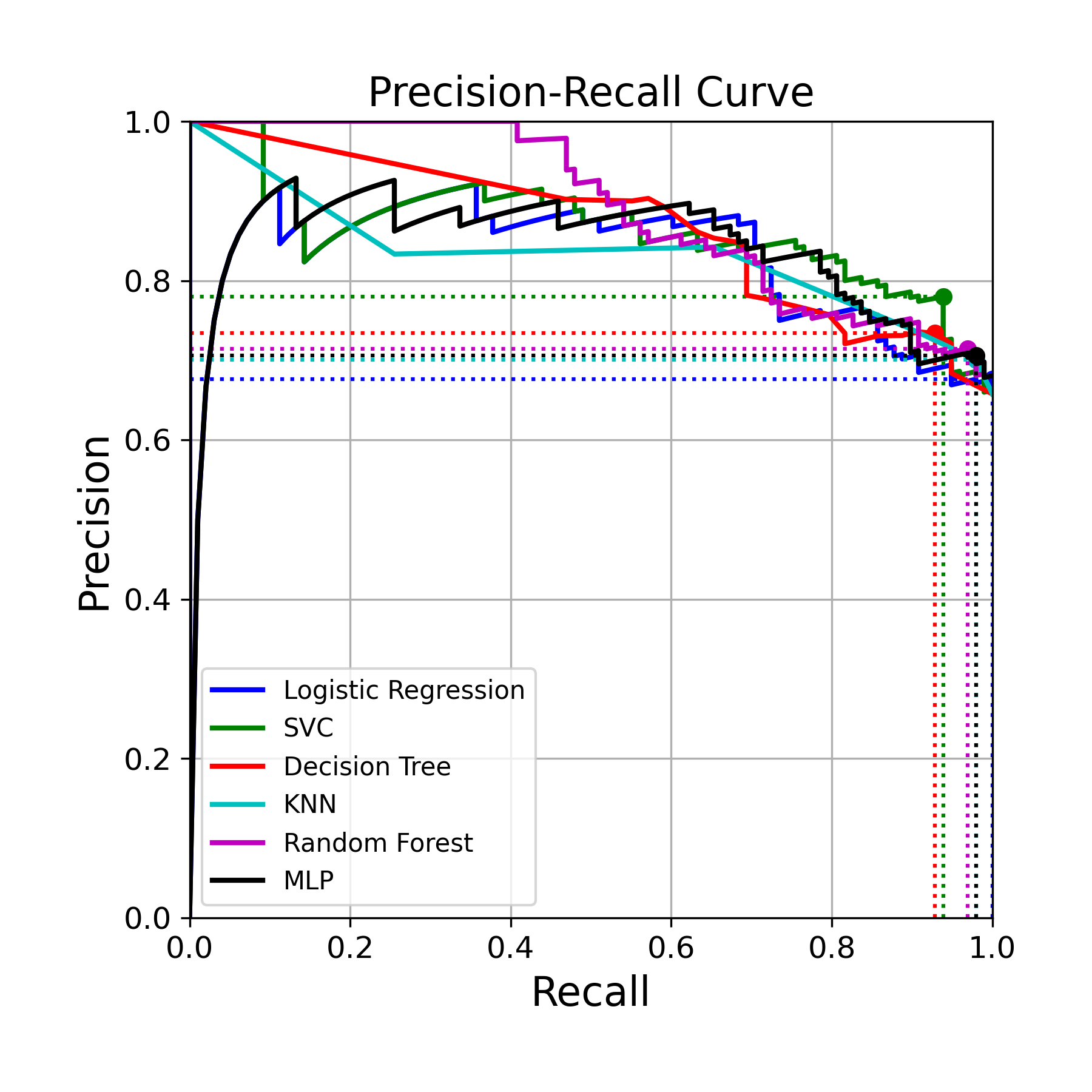

Supplement: S2 Fig — All training samples have been used except the ones identified as Tuberculous. The dots correspond to the points that maximize the F1 score. (TIF) [file pone.0259203.s002.tif]
