# Supplementary material for: Diagnostic accuracy of adenosine deaminase for pleural tuberculosis in a low prevalence setting: A machine learning approach within a 7-year prospective multi-center study
Source: PLoS One. 2021 Nov 4;16(11):e0259203. doi: 10.1371/journal.pone.0259203 (PMC8568264; doi:10.1371/journal.pone.0259203)
Supplement: S1 Table — The Random Forest classifier allows estimating the relative importance of each variable in the classification process. In view of the results, and in accordance with all the previously reported studies, in this classification process, “ADA” is the most important variable, followed by “age”. The rest of the variables are less useful, but we decided not to remove any of them a priori, but rather let each Machine Learning method assign a relative importance to each of them. (PDF) [file pone.0259203.s003.pdf]

S1 Table. Relative importance of features in the classification process. The Random Forest classifier allows estimating the relative importance of each variable in the classification process. In view of the results, and in accordance with all the previously reported studies, in this classification process, "ADA" is the most important variable, followed by "age". The rest of the variables are less useful, but we decided not to remove any of them a priori, but rather let each Machine Learning method assign a relative importance to each of them.

**Feature**	**Importance**
ADA	0.55
Age	0.14
Glucose	0.07
Proteins	0.06
MNC	0.05
RBC	0.03
Cell no	0.02
LDH	0.02
pH	0.02
