# Supplementary material for: Diagnostic accuracy of adenosine deaminase for pleural tuberculosis in a low prevalence setting: A machine learning approach within a 7-year prospective multi-center study
Source: PLoS One. 2021 Nov 4;16(11):e0259203. doi: 10.1371/journal.pone.0259203 (PMC8568264; doi:10.1371/journal.pone.0259203)
Supplement: S2 Table — (PDF) [file pone.0259203.s004.pdf]

S2 Table. Classification of patients by etiology.

ETIOLOGY (Subclassification)	N	%
**TUBERCULOSIS**	**44**	**19.1**
**MALIGNANCY**	**124**	**53.9**
Lung cancer	77	62.0604
Mesothelioma	15	12.0129
Lymphoma	10	8.0
Extrapulmonary cancer	22	17.7185
Breast cancer	8	6.472
Myeloma	2	1.6
Pancreatic cancer	1	0.8
Gastric MALT lymphoma	1	0.8
Ovarian cancer	3	2.4
Renal cell carcinoma	2	1.6
Pleuro-peritoneal serous carcinoma	1	0.8
Gastric cancer	1	0.8
Ewing sarcoma	1	0.8
Chronic lymphocytic leukemia	1	0.8
Esophageal cancer	1	0.8
**OTHER DIAGNOSES**	**62**	**26.8**
Uncomplicated parapneumonic pleural effusion	28	45.1
Uremic	2	3.2
Hemothorax	6	9.6
Pleural effusion after cardiac surgery cancer	7	11.2
Spontaneous bacterial pleuritis	1	1.6
Post-viral acute pleuro-pericardial disease	6	9.6
Systemic disease	3	4.8
Acute pulmonary embolism	5	8.0
Mesothelial atypical hyperplasia	1	1.6
Atypical lymphoplasmacytic hyperplasia	1	1.6
Meigs syndrome	1	1.6
Chylothorax	1	1.6
**TOTAL**	**230**	**100.0**
