# Supplementary material for: Diagnostic accuracy of adenosine deaminase for pleural tuberculosis in a low prevalence setting: A machine learning approach within a 7-year prospective multi-center study
Source: PLoS One. 2021 Nov 4;16(11):e0259203. doi: 10.1371/journal.pone.0259203 (PMC8568264; doi:10.1371/journal.pone.0259203)
Supplement: S3 Table — (PDF) [file pone.0259203.s005.pdf]

S3 Table. Methods and comparative diagnostic yield regarding tuberculous and malignant pleural effusions.

	TUBERCULOUS		MALIGNANT	
	N	%	N	%
**TOTAL**	**44**	**100.0**	**124**	**100.0**
**PLEURAL FLUID**	**44**	**100.0**	**124**	**100.0**
Positive acid-fast bacilli smear	1	2	0	0
Positive Lowenstein culture				
First pleural fluid sample	25	56	0	0
All pleural fluid samples	28	63	0	0
Positive PCR	5	11	0	0
Positive cytology				
First pleural fluid sample	0	0	63	50
All pleural fluid samples	0	0	69	55
**PLEURAL BIOPSY**	**21**	**47.7**	**47**	**37.9**
**CLOSED PLEURAL BIOPSY**	12	27.2	7	5.6
Positive Lowenstein culture	7	58	0	0
Positive PCR	6	50	0	0
Positive granuloma	11	91	0	0
Positive histopathology	0	0	7	100
**THORACOSCOPIC PLEURAL BIOPSY**	9	20.4	40	32.2
Positive Lowenstein culture	7	77	0	0
Positive PCR	6	66	0	0
Positive granuloma	9	100	0	0
Positive histopathology	0	0	39	98
**BRONCHIAL SPECIMENS**	**40**	**90.9**	**72**	**58.1**
**SPUTUM SAMPLE**	25	56.8	0	0.0
Positive acid-fast bacilli smear	2	8	0	-
Positive Lowenstein culture	13	52	0	-
Positive PCR	7	28	0	-
**BRONCHOALVEOLAR LAVAGE**	15	34.1	72	58.1
Positive acid-fast bacilli smear	4	27	0	0
Positive Lowenstein culture	10	67	0	0
Positive PCR	8	53	0	0
Positive cytology	0	0	26	36
**BRONCHIAL BIOPSY**	0	0	52	41.9
Positive histopathology	0	-	36	69
