# Supplementary material for: Diagnostic accuracy of adenosine deaminase for pleural tuberculosis in a low prevalence setting: A machine learning approach within a 7-year prospective multi-center study
Source: PLoS One. 2021 Nov 4;16(11):e0259203. doi: 10.1371/journal.pone.0259203 (PMC8568264; doi:10.1371/journal.pone.0259203)
Supplement: S4 Table — Logistic Regression (Logit). Support Vector Machine (SVC). Decision Tree (DT). K-Nearest Neighbors (KNN). Multi Layer Perceptron (MLP). (PDF) [file pone.0259203.s006.pdf]

S4 Table. Parameters of the classifiers Logistic Regression (Logit). Support Vector Machine (SVC). Decision Tree (DT). K-Nearest Neighbors (KNN). Multi Layer Perceptron (MLP).

Classifier	Parameters
Logit	'C': 0.1, 'penalty': 'l1', 'solver': 'liblinear',tol: 1e-4, fit intercept: True, intercept scaling:1, class weight:None, max iter:100,multi class:'auto',n jobs = None
SVC	'C': 0.001, 'coef0': 0.0, 'degree': 2, 'kernel': 'rbf', 'probability': True, gamma:'scale', shrinking=True, tol:1e-3, class weight:None, max iter:-1, decision function shape:'ovr', break ties:False
DT	'max depth': None, 'min samples leaf': 10,criterion: 'gini', splitter: best, min samples split:2, min weight fraction leaf: 0.0, max features: None, max leaf nodes = None, min impurity decrease: 0.0, min impurity split:0, class weight: None, ccp alpha:0.0
KNN	'n neighbors': 10, weights: 'uniform', algorithm:'auto', leaf size:30, p:2, metric:'minkowski',metric params:None
RF	'max depth': 3, 'min samples leaf': 1, 'n estimators': 50, 'n jobs': -1, criterion:'gini', min sample split:2, min weight fraction:0.0, max features:'auto',max leaf nodes: None, min impurity decrease:0.0, min impurity split:None, bootstrap: True, oob score:False, class weight: None, ccp alpha: 0.0, max samples: None
MLP	'alpha': 0.1, 'hidden layer sizes': (10,), 'solver': 'lbfgs', activation: 'relu',learning rate: 'constant', learning rate init:0.001, power t=0.5,max iter:200, shuffle:True,tol:1e-4,momentum:0.9, early stopping:False,max fun:15000
