# Supplementary material for: Diagnostic accuracy of adenosine deaminase for pleural tuberculosis in a low prevalence setting: A machine learning approach within a 7-year prospective multi-center study
Source: PLoS One. 2021 Nov 4;16(11):e0259203. doi: 10.1371/journal.pone.0259203 (PMC8568264; doi:10.1371/journal.pone.0259203)
Supplement: S5 Table — All training samples have been used except the ones identified as Tuberculous. Area under the curve (AUC), accuracy (Acc), sensitivity (SEN), specificity (SPF) and F1 score (F1) of all the classifiers using three different thresholds: 0.5, the one that maximizes the Youden index in the receiver operating characteristic curve and the one that maximizes the F1 score in the precision-recall curve. (PDF) [file pone.0259203.s007.pdf]

S5 Table. Validation results of the binary classification problem where the positive class is *Malignant* and the negative class is *Other*. All training samples have been used except the ones identified as Tuberculous. Area under the curve (AUC), accuracy (Acc), sensitivity (SEN), specificity (SPF) and F1 score (F1) of all the classifiers using three different thresholds: 0.5, the one that maximizes the Youden index in the receiver operating characteristic curve and the one that maximizes the F1 score in the precision-recall curve.

		**T = 0.5**					**T max Youden Index**					**T max F1 score**				
	AUC	T	Acc	SEN	SPF	F1	T	Acc	SEN	SPF	F1	T	Acc	SEN	SPF	F1
Logit	0.75	0.5	0.68	0.88	0.31	0.79	0.68	0.74	0.70	0.80	0.78	0.27	0.68	1.00	0.08	0.81
SVC	0.79	0.5	0.79	0.94	0.49	0.85	0.65	0.75	0.76	0.75	0.80	0.50	0.79	0.94	0.49	0.85
DT	0.76	0.5	0.69	0.77	0.55	0.77	0.87	0.68	0.59	0.86	0.71	0.36	0.73	0.93	0.35	0.82
KNN	0.74	0.5	0.72	0.90	0.39	0.81	0.80	0.69	0.65	0.76	0.74	0.40	0.71	0.98	0.20	0.82
RF	0.79	0.5	0.74	0.91	0.41	0.82	0.76	0.64	0.47	0.98	0.63	0.41	0.72	0.97	0.25	0.82
MLP	0.78	0.5	0.72	0.84	0.51	0.80	0.73	0.72	0.65	0.84	0.75	0.12	0.72	0.98	0.22	0.82
