# Supplementary material for: Diagnostic accuracy of adenosine deaminase for pleural tuberculosis in a low prevalence setting: A machine learning approach within a 7-year prospective multi-center study
Source: PLoS One. 2021 Nov 4;16(11):e0259203. doi: 10.1371/journal.pone.0259203 (PMC8568264; doi:10.1371/journal.pone.0259203)
Supplement: S6 Table — All test samples have been used except the ones identified as Tuberculous. Threshold (T), area under the curve (AUC), accuracy (Acc), sensitivity (SEN), specificity(SPF) and F1 score (F1) of all the classifiers, using the best thresholds found in the validation stage. (PDF) [file pone.0259203.s008.pdf]

**S6 Table. Test results of the binary classification problem where the positive class is** *Malignant* **and the negative class is** *Other***.** All test samples have been used except the ones identified as Tuberculous. Threshold (T), area under the curve (AUC), accuracy (Acc), sensitivity (SEN), specificity(SPF) and F1 score (F1) of all the classifiers, using the best thresholds found in the validation stage.

| | **T** | **AUC** | **Acc** | **SEN** | **SPF** | **F1** |
|---|---|---|---|---|---|---|
| Logit | 0.27 | 0.79 | 0.68 | 0.96 | 0.00 | 0.81 |
| SVC | 0.50 | 0.74 | 0.76 | 0.88 | 0.45 | 0.84 |
| DT | 0.36 | 0.86 | 0.84 | 0.92 | 0.64 | 0.89 |
| KNN | 0.40 | 0.66 | 0.73 | 0.92 | 0.27 | 0.83 |
| RF | 0.41 | 0.84 | 0.70 | 0.96 | 0.09 | 0.82 |
| MLP | 0.12 | 0.77 | 0.73 | 0.96 | 0.18 | 0.83 |
