# Supplementary material for: Diagnostic accuracy of adenosine deaminase for pleural tuberculosis in a low prevalence setting: A machine learning approach within a 7-year prospective multi-center study
Source: PLoS One. 2021 Nov 4;16(11):e0259203. doi: 10.1371/journal.pone.0259203 (PMC8568264; doi:10.1371/journal.pone.0259203)
Supplement: S8 Table — Accuracy (Acc), balanced accuracy (bAcc), weighted F1 score (wF1) and area under the curve using one-vs-rest strategy (AUC) of all classifiers using the test samples. (PDF) [file pone.0259203.s010.pdf]

S8 Table. Multiclass classifier scores (Tuberculous, Malignant and Other). Accuracy (Acc), balanced accuracy (bAcc), weighted F1 score (wF1) and area under the curve using one-vs-rest strategy (AUC) of all classifiers using the test samples.

	**Acc**	**bAcc**	**wF1**	**AUC**
Logit	0.78	0.76	0.78	0.86
SVC	0.78	0.74	0.77	0.85
DT	0.85	0.88	0.85	0.89
KNN	0.74	0.68	0.72	0.81
RF	0.80	0.74	0.77	0.87
MLP	0.78	0.76	0.78	0.84
