# Supplementary material for: Diagnostic accuracy of adenosine deaminase for pleural tuberculosis in a low prevalence setting: A machine learning approach within a 7-year prospective multi-center study
Source: PLoS One. 2021 Nov 4;16(11):e0259203. doi: 10.1371/journal.pone.0259203 (PMC8568264; doi:10.1371/journal.pone.0259203)
Supplement: Attachment — Submitted filename: response_to_reviewers.pdf [file pone.0259203.s011.pdf]

JOURNAL REQUIREMENTS:

- **1) Please ensure that your manuscript meets PLOS ONE´s style requirements, including those for file naming.**

  Thank you for the suggestion. We did so.

- **2) You indicated that you had ethical approval for your study. In your Methods section, please ensure you have also stated whether you obtained consent from parents or guardians of the minors included in the study or whether the research ethics committee or IRB specifically waived the need for their consent. Thank you for the comment.**

  There was only one minor patient included in the study. The informed consent, both for being included in the study and for performing the thoracocentesis, was signed by her parents and it is available upon demand.

- **3) One of the noted authors is a group or consortium (Gipuzkoa Pleura Group Consortium). In addition to naming the author group, please list the individual authors and affiliations within this group in the acknowledgements section of your manuscript. Please also indicate clearly a lead author for this group along with a contact email address.**

  Thank you for the suggestion. We proceeded as you suggested.

REVIEWER 1:

- 1) **Introduction: the definition of machine learning should be introduced**. Thank you for your comment. We absolutely agree. We added a definition of machine learning in the two last paragraphs of the Introduction section.

- 2) **The manuscript should be reported following the STARD guideline**. Thank you for your comment. We tested the checklist of STARD 2015 guidelines, and we have tried to confirm that all the below mentioned items are explained in the manuscript. It is important to remark that our approach does not modify the current clinical tests and procedures, but it incorporates mathematical methods to analyze the results obtained from such tests. Consequently, some of the items of the STARD guidelines do not perfectly fit with our contribution. Please find below the detailed explanation on each item:

Title or abstract:

1. Identification as a study of diagnostic accuracy using at least one measure of accuracy (such as sensitivity, specificity, predictive values or AUC): In the abstract, the best sensitivity, specificity and AUC obtained by machine learning approach is provided, both in the tuberculous/non tuberculous classification and in the tuberculous/malignant/other classification.

Abstract

2. Structured summary of study design, methods, results and conclusions (for specific guidance, see

STARD for Abstracts): The abstract fulfills such structure: "prospective study, diagnosis according to composite reference standard, pretest probability in our clinical setting, parameters included, six different machine learning classifiers tested for two different classifications: tuberculous/non tuberculous and tuberculous/malignant/other, results including number of patients, measures of accuracy of both classifications and conclusions".

Introduction

3. Scientific and clinical background, including the intended use and clinical role of the index test: The Introduction exposes all these items in detail (lines 2-33). Briefly: tuberculosis is a global concern, pleural tuberculosis is a common manifestation of extrapulmonary disease and is paucibacillary, so diagnosis is more difficult and constitutes a clinical challenge. Pleural biopsy (even by thoracoscopy) is the "gold standard diagnostic tool" but attempts to find another less hazardous way to obtain the diagnosis of pleural TB have been constant for many decades. ADA is the most cost-effective biomarker in pleural fluid but historically and following the Bayesian interpretation of its diagnostic accuracy is only admitted as a rule-out test in low prevalence scenarios.
4. Study objectives and hypotheses: Objectives and hypotheses are clearly exposed in the Introduction (lines 2-33). Following our previous retrospective study in 2012, we try to explore the limits of ADA when combined with other parameters as a rule-in test, in a low prevalence setting and following a Machine Learning approach. Our hypothesis is that diagnosis of pleural tuberculosis can be obtained with more reasonable accuracy than historically expected by the use of biomarkers like ADA in low prevalence settings when combined with other common parameters used in daily clinical practice. Secondly, the Machine Learning approach could have an added value to discriminate malignant effusions amongst those lymphocytic and exudative pleural effusions of non-tuberculous origin.

Methods

Study design

5. Whether data collection was planned before the index test and reference standard were performed (prospective study) or after (retrospective study): Data

collection was actually planned before the index test (ADA) was performed, as it can be derived from the Inclusion Material and Methods section (lines 61-77).

Participants

6. Eligibility criteria: The Inclusion Criteria paragraph fulfill this item (lines 78-83). Age over 16 years, *de novo* diagnosed pleural effusion, which should be lymphocytic and exudative, diagnosis according to composite reference standard, along with signed informed consent were the eligibility criteria. No patient was included without fulfilling all these criteria.
7. On what basis potentially eligible participants were identified (such as symptoms, results from previous tests, inclusion in registry): This item is explained in the Methods section (63-65). Eligible participants were identified amongst all those patients diagnosed with a pleural effusion and in whom a thoracentesis was performed.
8. Where and when potentially eligible participants were identified (setting, location and dates): This item is explained in the Methods section (lines 36-40 and 63-65). Potentially eligible participants were identified in any hospital in the Historical Territory of Gipuzkoa, from March 2013 to June 2020.
9. Whether participants formed a consecutive, random or convenience series: The abstract states that the patients were consecutively recruited. Participants formed a consecutive series of patients over 16 years, diagnosed *de novo* with lymphocytic and exudative pleural effusion, with diagnosis according to the composite reference standard and who signed the informed consent in the Historical Territory of Gipuzkoa from March 2013 to June 2020.

Test methods

10. A) Index test, in sufficient detail to allow replication: The index test is described in lines 63-65: ADA in pleural fluid, along with age and routine parameters also in pleural fluid: pH, glucose, protein, lactate dehydrogenase, total and differential red and white cell counts.

    10 B) Reference standard, in sufficient detail to allow replication: The reference standard is described in lines 69-72 and 125-137. Diagnostic criteria of tuberculous pleural effusion were as follows: positive culture or Xpert/RIF assay for MTB in pleural fluid, pleural biopsy or sputum, or evident granulomatous inflammation in pleural tissue. Diagnostic criteria of malignant pleural effusion were the presence of malignant cells in pleural fluid or in pleural tissue. Diagnostic criteria of paramalignant and parapneumonic effusion are also explicitly mentioned in the Material and Methods section (lines 129-132).

11 Rationale for choosing the reference standard (if alternatives exist): the microbiological or molecular identification of MTB in pleural fluid or pleural tissue, along with the histological identification of granuloma in pleural tissue, remain currently the gold standard diagnosis of CONFIRMED pleural tuberculosis. (reference 31st of the manuscript)

12a Definition of and rationale for test positivity cut-offs or result categories of the index test, distinguishing prespecified from exploratory. Currently the most widely accepted cut-off level of ADA for the daily clinical practice is 40 U/l as stated in lines 363-364. All five meta-analysis performed and included in the manuscript (references 34-38) were carried out using this cut-off level; and in our previous retrospective study published in 2012 we did it too. There is some evidence regarding the lower levels of ADA in pleural fluid in older patients diagnosed with pleural TB (reference 7th of the manuscript), but we considered it essential to perform our study following the same pattern as previous studies, in order to compare with them more accurately.

12b Definition of and rationale for test positivity cut-offs or result categories of the reference standard, distinguishing prespecified from exploratory: There is neither cut-off diagnostic level in the diagnosis of the reference standard of pleural tuberculosis nor in the diagnosis of malignant, paramalignant or parapneumonic pleural effusion.

13a Whether clinical information and reference standard results were available to the performers or readers of the index test: Yes, it was so, in daily clinical practice ADA value in pleural fluid was performed in the first hours after admission; subsequently, all the clinical information and reference standard results became and it was absolutely available for all the colleagues included in the group (lines 92-93).

13b Whether clinical information and index test results were available to the assessors of the reference standard: The answer would be the same as in 13a.

Analysis

14. Methods for estimating or comparing measures of diagnostic accuracy

Several metrics to compare the performance of the classifiers have been used: Accuracy, Sensitivity, Specificity and F1-score. All these metrics have been obtained by averaging the validation predictions in a 5-fold cross validation. Also 95% CI has been obtained for all these metrics.

15 How indeterminate index test or reference standard results were handled

In our case, we do not have a classification result that can be considered indeterminate. Classifiers always make a prediction, either: tuberculosis / no tuberculosis, or tuberculosis / tumor / other. That is, the classifiers always make a prediction consisting of one of the classes with which they have been trained based on their decision threshold.

16 How missing data on the index test and reference standard were handled: The flowchart in the Methods section (Figure 1) states that patients with missing data were excluded from the study.

17 Any analyses of variability in diagnostic accuracy, distinguishing prespecified from exploratory

As there are a limited number of samples, the patients were not subdivided into different groups, as such, to perform different tests. Instead, a cross-validation has been done. This has made it possible to obtain an indicator of the variability of the precision of the methods, which is shown through the confidence intervals in tables 2 and 3.

18 Intended sample size and how it was determined. Calculation of sample size was performed following the Epidat 3.1 Statistical Program. For a sensitivity of 95%, specificity of 90%, prevalence (pre-test probability) of 10%, significance level of 5%, power of 80% and precision of 5%, the minimum sample size was 200 patients. A 25% loss was estimated so final sample size was calculated at 250 patients. We included these data in the Material and Methods section of the manuscript (lines 138-141).

Results

Participants

19 Flow of participants, using a diagram: We included a flowchart in the manuscript (Figure 1).

20 Baseline demographic and clinical characteristics of participants: All these data are exposed in Table 1.

21a Distribution of severity of disease in those with the target condition: In our study we did not assess the severity of the disease, but the accuracy for the diagnosis of a disease. Severity of a given disease does not imply changes in the diagnostic accuracy of a given biomarker like ADA, so we consider this data as non-essential for our purpose.

21b Distribution of alternative diagnoses in those without the target condition: All this information is available in the Supporting Information Section, Table S2.

22 Time interval and any clinical interventions between index test and reference standard: The time intervals and the clinical interventions were those usual in the daily clinical practice for the diagnosis of the etiology of a pleural effusion.

Test results

23 Cross tabulation of the index test results (or their distribution) by the results of the reference standard: The confusion matrices included in the Results section fulfill this item (Figures 5-10).

24 Estimates of diagnostic accuracy and their precision (such as 95% CIs): The results (Tables 2 and 3) now include 95% CIs.

25 Any adverse events from performing the index test or the reference standard: there could not be any possible adverse event with the index test in this study (measuring level of ADA in pleural fluid along with age and other routine parameters). Regarding the reference standard, there was no adverse event reported with pleural biopsies performed (closed or by thoracoscopy).

Discussion

26 Study limitations, including sources of potential bias, statistical uncertainty and generalizability: We enumerate the limitations of our study in the last paragraph of the Discussion section (lines 466-478).

27 Implications for practice, including the intended use and clinical role of the index test: In our opinion and after viewing the results of our prospective study, the biomarker ADA combined with other parameters is more useful than expected and reported through last decades for the presumptive diagnosis of pleural tuberculosis in a low prevalence setting. Along with this and secondly, discrimination of most likely malignant pleural effusions constitutes an added value (abstract and lines 479-483)

Other information

28 Registration number and name of registry: Since we are presenting a prospective observational study and not a clinical trial, there is no registration number. Nonetheless, as we stated in the manuscript (lines 94-96), the protocol of this study was approved by the Clinical Research Ethics Committee of Euskadi, with record number 11/12).

29 Where the full study protocol can be accessed: The full study protocol (in Spanish and Basque language) was sent to the above-mentioned Clinical Research Ethics Committee of Euskadi. Although it is not publicly available, and despite the fact that all relevant details are exposed in the manuscript, full study protocol could be provided upon demand.

30 Sources of funding and other support; role of funders: This information is given after the main manuscript: " This study was supported by grant PI 2016111036 from the Office of Health Research and Innovation, Department of Health, Government of the Basque Country, Spain" (lines 501-502).

- 3) **A flowchart should be used to depict the patient selection process**. Thank you for the comment. We do agree. We included a flowchart (Figure 1) regarding the patient selection process in the manuscript.

- 4) **The discussion section should be shortened, and the readability needs to be improved**. Thank you for the comment. We removed some paragraphs in the Discussion section (for instance the probably extensive comments on our previous and retrospective study from 1998 to 2008 and the paragraph regarding the inclusion of parapneumonic effusions); along with this, we transferred the explanation of the confusion matrices to the Results section, just next to Figures 6 and 7, in order to facilitate their understanding. The Discussion section actually improved in readability with these changes in our opinion.

- 5) **Two studies have investigated the clinical utility of machine learning approaches for TPE diagnostics (Respir Res 2019 Oct 16; 20 (1):220; Comput Methods Programs Biomed 2018 Jan; 153: 211-225). These two studies should be mentioned, and the strengths of the current study should be discussed**. Thank you very much for the suggestion. We actually knew only one additional study in this regard (reference 57: Seixas JM, et al. Artificial neural network models to support the diagnosis of pleural tuberculosis in adult patients. Int J Tuberc Lung Dis 2013; 17 (5): 682-686), but we decided not to include it because we were not able to view its relationship with the Machine Learning approach. We included those three studies and we analyzed the main differences with ours in the manuscript (lines 405-413): all the three studies are performed in a high-very high prevalence scenario, and patients without diagnosis according to composite reference standard are included in all of them.

- 6) **Parameters used in machine learning algorithms should be reported.**
  Thank you for the comment. The parameters used by the classifiers have been included as supplementary material, and referenced in the Results section.

- 7) **95%CI should be added to each table.**
  Thank you very much for the comment. We did so.

- 8) **Line 348: is this a retrospective study?** No, It is not**.** This current study is prospective. As you can see, in line 348 (of previous manuscript) we were talking about our previous retrospective study published in 2012 in PLoSONE. Anyway, as we said when answering question 4th, we shortened substantially the paragraph dedicated to this theme in the Discussion section.

- 9) **The term "validation dataset" should be modified as "training dataset".**

Thank you for the comment. It is true that we have not used a validation set as such. However, when the term "validation set" is used in the document, we are not referring to the training set, but to the validation predictions in the cross-validation. In cross-validation, the training set is divided into k subsets. The models are then trained with

samples from k-1 subsets and validated with the rest. This operation is done k times with different validation subsets and the results are averaged. References to the “validation set” have been removed in the text, as it can lead to confusion, and the sentences where it appeared have been rewritten to refer to the “validation predictions”.

- 10) **The major limitation of this study is the small sample size.**

Thank you for the comment. As we state in the manuscript, the number of lost cases was lower than estimated and the minimum required sample size of 200 patients was reached, so diagnostic ability was not diminished (lines 138-141 and 472-475).

- **11) Methods: the software and packages used for machine learning should be reported.**

Thank you for the comment. A sentence describing the software used has been added in the statistical analysis section, after naming the types of classifiers that have been used (lines 161-165).

REVIEWER 2:

- 1) **The title is inappropriate as several other parameters besides ADA were used to train the algorithm for machine learning**. Thank you for the comment. Your observation is not meaningless, but in our study we decided to name it in this way due to two main reasons:
    - ADA in pleural fluid was the most important parameter when predicting the diagnosis of TPE. That was our base hypothesis, and it was validated during the study (the Random Forest classifier calculated its relative importance in the classification process at 0.55, while the next variable was age, with 0,14).
    - Our hypothesis is, as mentioned in the manuscript, that THE ADDITION OF OTHER PARAMETERS TO ADA in pleural fluid could lead us to obtain a suitable diagnostic accuracy for TPE. In this sense, ADA in pleural fluid would remain the diagnostic tool core. In our previous study in 2012 we added the lymphocyte proportion to ADA in pleural fluid, and in this current study we add all the other routine parameters in pleural fluid along with age; and we switch to a machine learning approach.

- 2) **The notion that pretest probability remained 3.8% during the entire study period is incorrect. The denominator for this assumption includes all pleural effusions, while authors studied only lymphocytic exudative effusions. During the study period, the entire province had only 45 patients of tuberculous pleural effusion. 44 of these were included in the present study. This appears to be a biased sample since all the lymphocytic exudative pleural effusions in the**

province don´t appear to have been included. Thank you for the comment. Should we state that "pre-test probability" (most authors name it "prevalence" when writing about TPE) refers to the number of cases of a given type of pleural effusion divided by the total number of pleural effusions studied through a period of time and in a given clinical setting. In this sense, the pre-test probability that a patient diagnosed with pleural effusion in the clinical scenario where you work suffers from a TPE would depend on these variables. In the Historical Territory of Gipuzkoa and from March 2013 to June 2020 a total of 1177 pleural effusions were diagnosed, and only 45 out of them were of tuberculous origin (44 of lymphocytic cellularity). This results in a pre-test probability of 3.8%.
We obviously DO NOT manage or diagnose absolutely all the pleural effusions in our Historical Territory, but we actually did so with all those cases of TPE; and we confirmed our database in this sense with official data from the Basque Health System and the Tuberculosis Control Program implemented in 2003 and with high reliable data collection since 1995. In regard to non-tuberculous pleural effusions, we included 186 consecutive cases of lymphocytic (due to limited funding) and exudative pleural effusion in whom diagnosis according to composite reference standard was obtained. Many other patients were excluded due to multiple reasons, all of them listed in the flowchart of the participants given in the manuscript.

- 3) **Patients where no definitive diagnosis could be reached were excluded, as were patients with any disease that could clearly explain the effusion. This should no have been done. Rather they could have used as the validation set for checking the accuracy of the algorithm**. Thank you for the comment. First, should we state that in this prospective study we tried to amend the deficiencies of our previous study published in 2012: all patients included should have diagnosis according to composite reference standard.

Furthermore and with regard to including patients with other previously diagnosed pathologies that could explain the effusions in the validation group, we decided not to do so in order to validate our methods under the same conditions in which they were trained. That is, with patients selected with the same criteria. Only *de novo* diagnosed pleural effusions were included. The reason was obvious: when a patient previously diagnosed with lung cancer is admitted to the hospital with a pleural effusion, physicians in daily clinical practice tend to assume that such effusion is malignant after discarding other etiologies, and diagnosis according to composite reference standard may not be the rule. We decided to analyze the diagnostic accuracy of ADA + age + routine parameters + Machine Learning for *de novo* diagnosed pleural effusions and with gold standard diagnosis.
However, the suggestion made by the reviewer could be a starting point for a different study as the one intended in this work: analyzing the performance of the

presented methods in a group of patients without gold standard or without a definitive diagnosis.

- 4) **I am still not sure whether the complicated machine learning algorithm actually improved diagnostic accuracy or not. Firstly, there is no clarity on why a particular set of variables were selected to train the algorithm. It is quite possible that less or more have been better. One cannot decipher the contribution of each parameter towards the overall decision-making. In particular, the overall sensitivity of the algorithm for lymphocytic exudates was lower than that of ADA, but the specificity was higher. The same result could have been easily obtained by shifting the arbitrarily defined threshold of 40 U/l to another value. Authors need to describe the diagnostic accuracy of ADA at different thresholds. For a confirmatory test, high specificity and acceptable sensitivity are needed, and this can be easily achieved using ADA alone**.

Thank you for the comment. With respect to the set of variables selected to train the models, the Random Forest classifier allows estimating the relative importance of each variable in the classification process. By doing this analysis, the following results were obtained.

ADA: 0.55
Age: 0.14
Glucose: 0.07
Proteins: 0.06
MNC: 0.05
RBC: 0.03
Cell no.: 0.02
LDH 0.02
pH 0.02

In view of the results, and in accordance with all the previously reported studies, in this classification process, “ADA” is the most important variable, followed by “age”. The rest of the variables are less useful, but we decided not to remove any of them a priori, but rather let each Machine Learning method assign a relative importance to each of them.

This explanation has been added as supplementary material referenced in the Statistical Analysis section.

In order to compare the obtained classification results with a classification method that used the ADA alone, we performed the same experiments with the ADA, that is, we obtained three thresholds: default threshold (in this case 40 U/l), the threshold that maximized the Youden index in the ROC curve, and the threshold that maximized the f1-score in the PR curve. It is interesting to note that the threshold that maximized the youden index with our samples was 40 U/l as well. The threshold that maximized the f1 score in the PR curve was 43 U/l. With these thresholds two set of metrics were extracted:

```
Threshold=40.00 Acc 0.93 SEN 0.97 SPC 0.93 | Youden
Threshold=43.00 Acc 0.95 SEN 0.94 SPC 0.95 | F1-score
```

Better Sensitivity/Specificity are obtained with other methods. For example, Logistic Regression gets 0,97/0,94 (youden 0.91). SVC and MLP gets 0,94/0,97 (youden 0.91). Also, better Accuracy is obtained with SVC (0,97) and MLP (0,96).

Along with all the above mentioned explanations and following the Bayesian analysis of the data, it is evident in the pre-post test probability curve (Figure 11) that addition of all the variables included in the study increased substantially and mostly the Positive Predictive Value in a low prevalence scenario.

As a final comment in this regard, another advantage of having trained ML models compared to using the ADA> 40 U/l is that we can make the distinction between tuberculosis, tumor and others.

This explanation has been added to the “Performance of the classifiers versus ADA alone” section at the end of the Results section (lines 321-345).

- 5) **The meaning and interpretation of the figures on ROC curves, precision-recall curves, and confusion matrices is not clear to me, not it has been adequately explained. I am not an expert in machine learning techniques, and neither is most of the readership of this journal.**

Thank you for the comment. Two new references have been added describing both ROC and PR curves. The explanation of what the rows and columns of a confusion matrix represent is in the Results section, after they are mentioned for the first time (lines 260-268). We hope that these descriptions and references help to understand the concepts of ROC and PR curves, and confusion matrices as well. We consider that they are sufficiently extended concepts, and that given the cross-sectional and multidisciplinary nature of the journal, its readers will have no problems understanding them.
