# Supplementary material for: Diagnostic accuracy of adenosine deaminase for pleural tuberculosis in a low prevalence setting: A machine learning approach within a 7-year prospective multi-center study
Source: PLoS One. 2021 Nov 4;16(11):e0259203. doi: 10.1371/journal.pone.0259203 (PMC8568264; doi:10.1371/journal.pone.0259203)
Supplement: Attachment — Submitted filename: Response to reviewer.pdf [file pone.0259203.s012.pdf]

Reviewer #1: In this study, the authors investigated the diagnostic accuracy of conventional biomarkers for TPE. The strength of this study is machine learning analysis, which has been proved to be a valuable method to improve the diagnostic accuracy of biomarkers. I have some comments.

Thank you for your comments. Please find below a point by point reply.

1. The current version of the manuscript is too long. The readability of the manuscript is poor. I strongly suggest authors shorten the length of the manuscript to make it more clear and focus. The current version of the manuscript is entirely unacceptable.

We regret that the reviewer found the manuscript difficult to read. Due to the nature of the contribution of the manuscript and the scope of the journal, we are aiming at three different groups of potential readers: clinicians, clinicians with solid training in statistics, and mathematicians/statisticians. We have tried to achieve a delicate balance between the depth provided to technical details and the clarity required to make our contribution accessible to clinicians who may be only interested in the application. A thorough explanation of technical details is unavoidable both to enhance the transparency of the methods and results, and to allow replicability.
Despite of all previously mentioned, we have done our best to make the manuscript easier and shorter whenever possible.

2.Figures 5, 6, 7, 8, 9, and 10 should be deleted because sensitivity and specificity have been reported in Table 2 and Table 3. In machine learning analysis, it is meaningless to report the results of the training cohort.

Thank you for your comment. Following your recommendation we have removed these figures.

3. AUC should be reported in table 2.

Thank you for your comment. Following your recommendation we have included the area under the curve in Table 2.
